# Pain and satisfaction during rigid cystoscopic ureteral stent removal: a preliminary study

**DOI:** 10.1186/1471-2490-14-90

**Published:** 2014-11-18

**Authors:** Jae Heon Kim, Sun Young Park, Mun Gyu Kim, Hoon Choi, Dan Song, Sung Woo Cho, Yun Seob Song

**Affiliations:** Department of Urology, Soonchunhyang University Hospital, 657 Hannam-Dong, Yongsan-Gu, Seoul 140-743 Korea; Department of Anesthesiology and Pain Medicine, Soonchunhyang University Hospital, Seoul, Korea; Department of Urology, Korea University Hospital, Ansan, Korea; Department of Surgery, Soonchunhyang University Hospital, Seoul, Korea

**Keywords:** Stent, Stent exchange, Stent removal, Cystscopy, Urolithiasis, Pain

## Abstract

**Background:**

Cystoscopy evokes discomfort and pain, especially in males. The cystoscopic retrograde approach is standard in the removal of ureteral stents. However the satisfaction and degree of pain during the procedure according to the use of several pain controlling methods are unclear.

**Methods:**

This is a cross-sectional survey of 60 patients who underwent cystoscopic ureteral stent removal under intravenous analgesics (group 1, n = 20), midazolam induction (group 2, n = 20), and propofol (group 3, n = 20). Procedural pain and post-procedure satisfaction were determined, and cost differences between the approaches were clarified.

**Results:**

Group 2 and 3 showed significantly less pain than group 1 (P < 0.001) and significantly higher satisfaction rate than group 1 (P < 0.001). Comparison of groups 2 and 3 revealed showed significantly less pain and higher satisfaction rate in group 3 (P < 0.001 for both). In Group 1, 17 (85.0%) patients wanted other treatment modalities, compared to eight group 2 patients (40.0%) and no group 3 patients.

**Conclusions:**

Considering the potential pain and dissatisfaction of rigid cystoscopic ureteral stent removal, procedures utilizing moderate sedation with midazolam or general anesthesia using propofol without muscle relaxation should be considered.

**Trial registration:**

KCT0001260.

**Electronic supplementary material:**

The online version of this article (doi:10.1186/1471-2490-14-90) contains supplementary material, which is available to authorized users.

## Background

Although the real benefit of ureteral stenting after ureteroscopic removal of stone (URS) is contentious, the chance with ureteral stenting following ureteroscopic removal of stone is frequent [1]. After URS, ureteral stents are removed at post-operative 1 or 2 weeks, typically by cystoscopic retrograde removal [2, 3]. However, because of the rigidity and larger diameter of cystoscopes, most patients need analgesia and some patients need deep sedation during the procedure [2, 3]. In real practice, those stents are removed mostly in the outpatient setting using urethral lubrication jelly with or without narcotic intramuscular premedication [4–6].

Recently, lubrication jelly and lidocaine injection were reported to be no more effective for pain control during cystoscopy [7–11]. A flexible cystoscopy is a good alternative to rigid cystoscopy to reduce pain during procedure, but flexible cystoscopy is less available in korea and moreover there have been little reports about ureteral stent removal with flexible cystoscopy.

There have been many studies about pain during rigid or flexible cystoscopy, but there have been few studies about pain during cystoscopic stent removal. Although shorter in duration than cystoscopy, cystoscopic stent removal yields a similar pain to cystoscopy, and moreover larger diameter of rigid cystoscopy is needed for use of foreign body forceps. In our previous pilot study, cystoscopy using midazolam produced marginally greater satisfaction among men [6]. This is the main reason why we adapted diverse pain controlling method including propofol. The aim of this prospective, randomized, pilot study was to compare the satisfaction about cystoscopic stent removal according to different pain relief methods and to compare the costs.

## Methods

### Study sample

From September 2012 to March 2013, 60 male patients with a history of prior URS and ureteral stenting due to ureteral stone were enrolled. Informed consent was obtained from all patients. The mean age of patients was 47.45 years. Subjects with severe cardiovascular disease, pulmonary disease, liver disease, and drug abuse history were excluded as were patients with a prior cystoscopy procedure were excluded. The 60 patients were sub-classified randomly according to several pain controlling methods: cystoscopy + intravenous (IV) analgesics (group 1, n = 20); cystoscopy + midazolam (group 2, n = 20); and cystoscopy + propofol (group 3, n = 20). This study was approved by Institutional review board of Soonchunhyang University Hospital. ***Trial registration*** KCT0001260.

### Procedures

All patients were placed in the dorsolithotomy position in the operation room. The same two urological surgeons (Jae Heon Kim and Yun Seob Song) performed all cystoscopic ureteral stent removals using a 17.5 Fr rigid cystoscope. Prior to the procedure, the urethra was instilled with 2% lidocaine topical jelly. After 5–10 min, the cystoscope was introduced to the urethra and bladder, and the ureteral stent was removed using foreign body forceps. In the operating room, electrocaridography, noninvasive blood pressure monitoring, and pulse oximetry monitoring were done. Vital signs were checked during the procedure and after the procedure in the day care unit. The presence of complications including oxygen desaturation, autonomic movement, arrythmia, injection pain, and phlebitis were also examined. Before discharge, the patients were asked to rate their comfort level using a visual analog scale (VAS) and satisfaction scale, detailed in the Additional file 1. Recovery from sedation was assessed by the mini-mental state examination (MMSE).

### IV administration of ketorolac

Intravenous analgesic administration was performed after lidocaine jelly instillation into the urethra. Intravenous administration of ketorolac 30 mg was used for pain control. Before discharge, the patients were asked to rate their comfort level as described above.

### Moderate sedation with midazolam

Midazolam with doses of 3-5 mg (no more than 0.03 mg/kg) was administrated to the subjects after lidocaine jelly instillation. The status of sedation was measured and divided according to five stages, as described in the Additional file 1. Cystoscopy was started when the stage was over three. After the procedure was finished, the midzolam antidote, flumazenil was administrated.

After the procedure, the patient was transferred to day care unit and was discharged when they displayed normal orientation of time and space with vital signs within the normal range.

### Deep sedation with propofol

Patients received an injection of 0.2 mg glycopyrollate about 20 min before induction of deep sedation. Sedation was induced with propofol 2 mg/kg without muscle relaxation and was maintained using propofol 10 mg/kg/h. After induction, the anesthesiologist applied a face mask and assisted with ventilation with 100% O_2_. After the procedure, the patient was transferred to day care unit and was discharged when they displayed normal orientation of time and space with vital signs within the normal range.

### Cost calculation

Cost was described as medical insurance fee and real patient expense. In Korea, due to National Medical Insurance system, a patient may pay 20-100% of total medical insurance fee. Rate of exchange between Korea Won and the US dollar was 1120.6 won for 1 dollar.

### Treatment satisfaction

The treatment satisfaction questionnaire included five subscales: “very satisfied”, “satisfied”, “average”, “not satisfied”, and “totally not satisfied”. These subscales were divided into two groups: “Satisfactory” included “very satisfied” and “satisfied”, and “Not satisfactory” included “average”, “not satisfied”, and “totally not satisfied”.

### Questionnaire about seeking another method

After the procedure, a questionnaire solicited responses about seeking other pain controlling method. The question asked was “Do you prefer another pain controlling method if it were effective although you could pay more?”

### Statistical analyses

Data were analyzed using SAS version 9.1 (SAS Institute, Cary, NC, USA). The Kolmogorov-Smirnov test was used to verify the normality of distribution of continuous variables. Nonparametric tests of comparison were used for variables evaluated as not normally distributed. Median and minimal to maximal range were used as appropriate to describe statistics. Difference testing between groups was performed using Kruskal-Wallis test and Mann–Whitney test as appropriate.

## Results

There was no significant difference among blood pressure, pulse rate, O2 saturation during the procedure including those 3 different methods. The differences of pre-operative and post-operative MMSEs including pre-operative and post-operative were not noted among the three groups. The time duration including procedural time and recovery time showed longer in group 2 and 3 than group1 (Table 1).

**Table 1:** **Satisfaction and pain scores among the three groups**

	Group 1	Group 2	Group 3	P value
Age	49.50 (26–70)	47.50 (15–70)	49.50 (29–72)	0.731
BMI	26.5 (19.8-31.2)	25.6 (21.4-28.3)	26.0 (22.6-29.4)	0.361
Time duration (min)	11.3 (8.6-30.5)	32.6 (29.6-40.5)	50.4 (45.3-75.4)	0.021
Duaration of procedure (min)	2.3 (1.5-3.3)	2.4 (1.3-3.4)	2.2 (1.0-3.2)	0.243
VAS	8.00 (6–10)^*,†^	5.00 (1–7)^†,‡^	0.00 (0–1)^*,‡^	<0.001
Satisfaction	1 (0–3)^*,†^	3 (1–5)^†,‡^	5 (4–5)^*,‡^	<0.001
Willing to undergo the procedure (VAS)	2 (0–4)^*,†^	5 (4–8)^†,‡^	7 (6–9)^*,‡^	<0.001

*Group 1*, Cystoscopy + IV analgesics, *Group 2*, Cystoscopy + midazolam, Group 3, Cystoscopy + propofol, *BMI* Body mass index, *VAS* Visual analog pain scale.

*Time duration* procuedural time + recovery time.

Data are expressed as median number with minimum to maximum number.

P values were analyzed by Kruskal-Wallis test.

^*,†,‡^:significant differences by Post hoc analysis.

**Figure 1:**
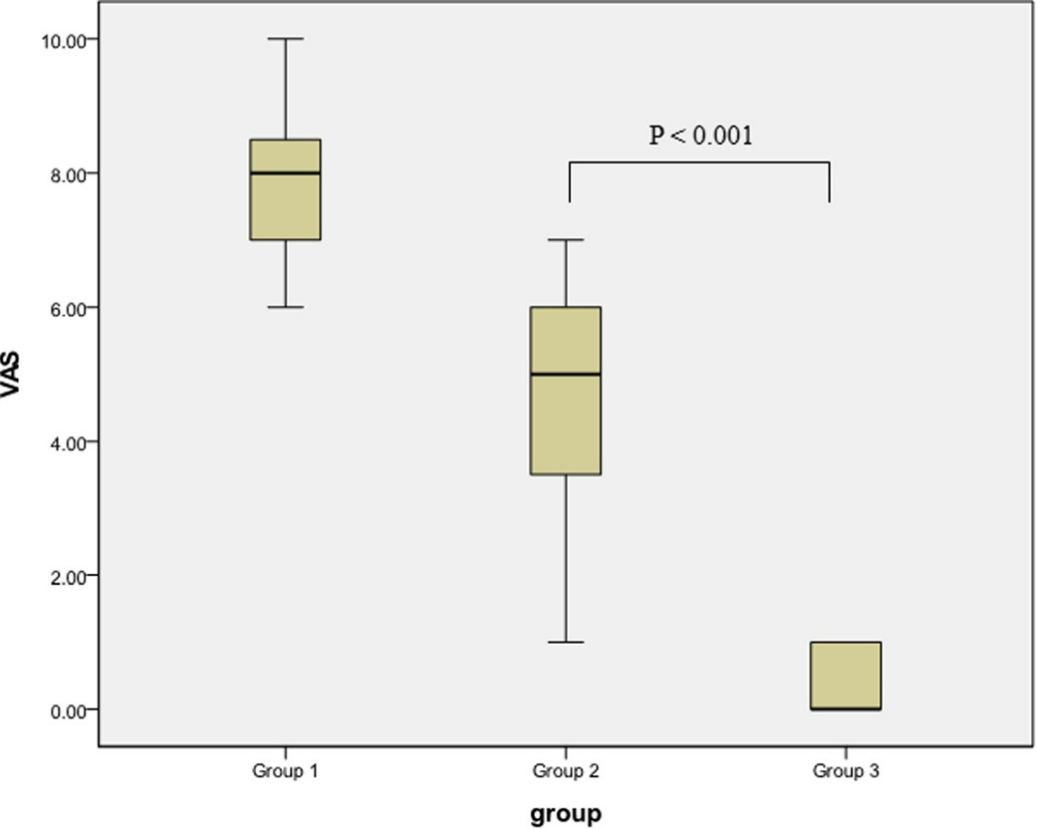
**Comparison of VAS among group 1 (cystoscopy + IV analgesics), group 2 (cystoscopy + midazolam), and group 3 (cystoscopy + general anesthesia using propofol).**

**Figure 2:**
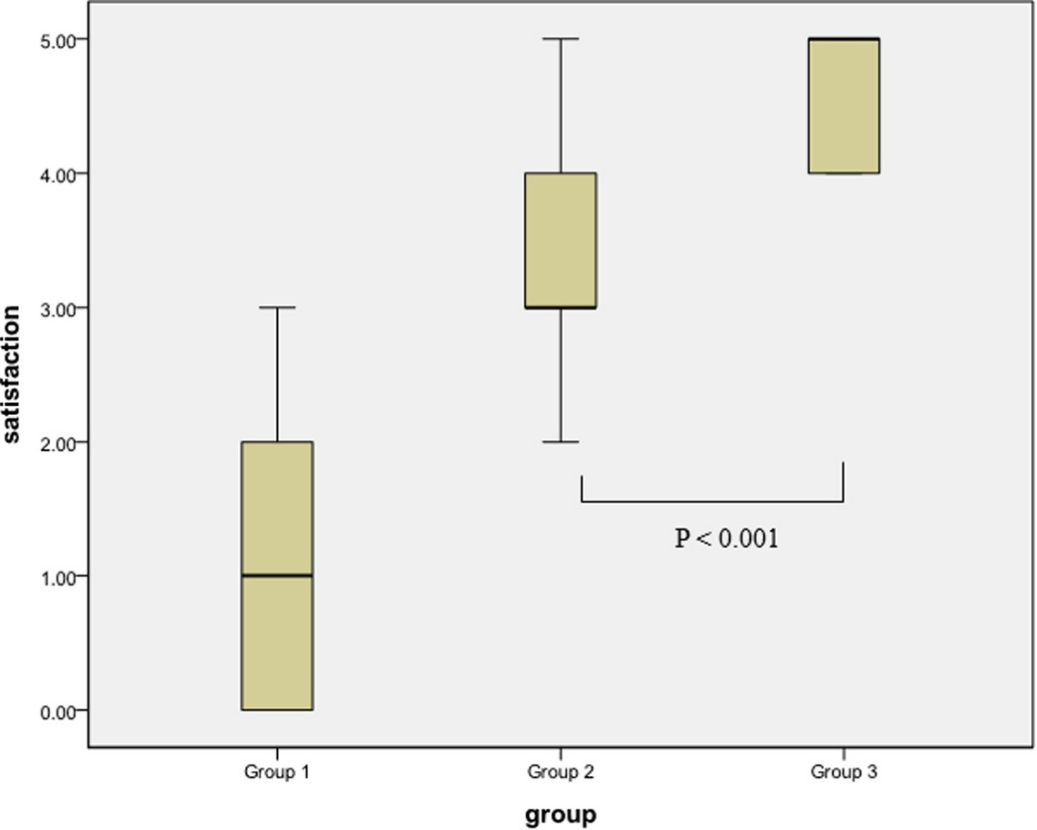
**Comparison of satisfaction among group 1 (cystoscopy + IV analgesics), group 2 (cystoscopy + midazolam), and group 3 (cystoscopy + general anesthesia using propofol.**

Group 1 experienced more pain and more dissatisfaction with the procedure than group 2 and group 3. VAS of group 1 was higher than that of group 2 and group 3 (P <0.001) (Table 1). Satisfaction scale of group 1 was lower than that of group 2 and group 3 (P <0.001) (Table 1). Comparison of group 2 and 3 revealed lower VAS in group 3 (P <0.001) and higher satisfaction rate in group 3 (P < 0.001) (Figures 1 and 2).

Total medical insurance fee for group 1, 2, and 3 was US102.63, US108.65, and US218.47, respectively (Table 2). For real patient expense, the cost in the same respective order was US57.94, US61.55, and US119.98 (Table 2). Detailed expenses are provided in Table 2.

**Table 2:** **Cost expenses among the three groups**

	Group 1		Group 2		Group 3	
	Cost (won)	Patient expense (won)	Cost (won)	Patient expense (won)	Cost (won)	Patient expense (won)
Fee for procedure	75,647	45,388	75,647	45,388	75,647	45,388
Consultation fee	13,090	13,090	13,090	13,090	13,090	13,090
Normal saline 1 L	1,099	659	1,099	659	1,099	659
Intravenous injection fee	3,315	663	3,315	663	6,630	1,326
Day care unit					38000	38000
Aneshesia fee					92,950	18,590
Profopol					17,398	17,398
Midazolam			761	456		
Flumazenil			7,868	4,720		
IV NSAIDs	1,889	1,134				
Blood O2 saturation monitoring	5,490	1,098	5,490	1,098		
ECG monitoring	6,460	1,292	6,460	1,292		
Blood pressure monitoring	8,020	1,604	8,020	1,604		
Total costs (won)	115,010	64,928	121,750	68970	244,814	134,451
Total costs (US dollar)	102.63	57.94	108.65	61.55	218.47	119.98

*Gourp 1* Cystoscopy + IV analgesics, *Group 2* Cystoscopy + midazolam, *Group 3* Cystoscopy + propofol, *ECG* electrocardiography, *NSAIDS* non-steroidal anti-inflammatory drugs.

Comparison of groups 2 and 3 revealed less pain and higher satisfaction rate in group 3 (P <0.001 for both). In group1, 17 (85%) patients wanted other treatment modalities, whereas in eight of group 2 patients (40%) and no group 3 patients wanted other treatment modalities. Group 1 revealed lower VAS score of willing to undergo the procedure again than group 2 and group 3 (<0.001). Group 2 showed also lower VAS score of willing to undergo the procedure again than group 3 (<0.001) (Table 1).

## Discussion

Cystscopy is the standard technique used to removal or exchange a ureteral stent. In addition to the large diameter of cystoscopies, which can induce pain, several conditions make this technique more difficult, especially in male patients, due to the longer urethra and prostatic enlargement.

Several retrograde methods without conventional cystoscopy have been developed [7–10]. Successful outcomes have been reported using retrograde ureteral stent removal or change under fluoroscopic guidance, but most patients in these studies were female, and only one study included male patients [10].

Ureteroscopy is one of the most common methods to treat urinary stones [11]. In many cases, ureteral stent insertion follows ureteroscopy [1]. Although cystoscopic ureteral stent removal is common, discomfort associated with the procedure is unclear. Our study is the first clinical trial to address this issue.

Local anesthesia has long been used in men undergoing rigid cystoscopy. Recent reports indicated that lidocaine gel has no effect on pain during cystoscopy [7–11]. The diverse efficacy of lidocaine gel may be because the absorption of topical lidocaine is slow and incomplete. Several groups have demonstrated that maximal lidocaine absorption requires 15 to 60 minutes [5, 12].

To overcome this limited effect of lidocaine jelly, several methods have been introduced such as sleep induction using midazolam, pain killers, or listening to music [4, 6, 13]. Midazolam is a well-known sedative drug with amnesic properties. Previous studies have demonstrated that midazolam can yield anterograde amnesia without retrograde amnesia [14–17]. Midazolam produces the immediate onset of anterograde amnesia in patients, which could be useful in forgetting the painful events [14].

One of the prominent features of our study was that, for the first time, we adapted a propofol in cystoscopy or cysoscopic ureteral stent removal. Propofol is safe and effective during gastrointestinal endoscopy procedures [18, 19]. Moreover, it has been associated with shorter recovery time, better sedation, and lack of a harmful effect on cardiopulmonary function. Our study showed that both the group with midazolam and propofol showed longer time duration but the differences were not large. Considering the nature of pilot study to use propofol, we had assistance of anesthetic department for safety. In the future, the procedures using propofol might be feasible in outpatient department.

In this study, the satisfaction was the greatest in the group with using propofol. Cystoscopic procedure with IV pain killers was not effective at all. Procedures using midazolam yielded less pain and greater satisfaction than procedures with IV pain relievers. Patients treated with propofol reported the greatest satisfaction despite spending additional recovery time in the day care unit.

Moreover, the gap of real expense among the three groups was not large. This is might be due to a unique medical insurance system in Korea. The gap difference of cost should be validated in other countries with different medical systems.

The present study has several limitations. We did not assess the pain felt by patients during each step of the procedure. Moreover, the sample size was relatively small, and the study was not blinded for patients and physicians, which could result in some bias in data interpretation or reporting of satisfaction and pain levels. Second, the sample size was relatively small but owing to its nature of pilot study, the differences of main outcomes among each groups were definite.

## Conclusions

Urologists have to pay more concern to cystscopic ureteral stent removal. With the traditional methods of lidocaine jelly and pain killers, patients have to suffer from pain and discomfort. Midazolam and propofol could be a options to control both. Considering the safety and the high prevalence of use of midazolam and propofol, urologists should not hesitate to adapt new methods in pain control during cystoscopic ureteral stent removal.

## Electronic supplementary material

Additional file 1: **Mini-mental state examination.** (DOCX 15 KB)
