# Supplementary material for: Pain and satisfaction during rigid cystoscopic ureteral stent removal: a preliminary study
Source: BMC Urol. 2014 Nov 18;14:90. doi: 10.1186/1471-2490-14-90 (PMC4242475; doi:10.1186/1471-2490-14-90)
Supplement: Supplementary file 1 — Additional file 1: Mini-mental state examination. (DOCX 15 KB) [file 12894_2014_382_MOESM1_ESM.docx]

Additional file 1

1. Sedation scale: Alert (0); Drowsy, partial lid closure (1); Eyes closed, responding to verbal command (2); Unresponsive to verbal command, but responsive to pain (3); Unresponsive (4).

2. Visual analog pain scale

1 2 3 4 5 6 7 8 9 10

3. Recovery test (mini mental state examination)

- Orientation for time (five points): what is the (year) (month) (date) (day) (season)?
- Attention and calculation (five points): Serial 7’s. One point for each correct. Stop after five answer.
- Language (three points): follow a three-stage command
- Take a paper in your right hand, fold it in half, and put it on the floor.

One point is given for each portion, with a maximum total of 13 points.
